# Differences in Salivary Proteins as a Function of PROP Taster Status and Gender in Normal Weight and Obese Subjects

**DOI:** 10.3390/molecules26082244

**Published:** 2021-04-13

**Authors:** Melania Melis, Mariano Mastinu, Stefano Pintus, Tiziana Cabras, Roberto Crnjar, Iole Tomassini Barbarossa

**Affiliations:** 1 Department of Biomedical Sciences, University of Cagliari, Monserrato, 09042 Cagliari, Italy; melaniamelis@unica.it (M.M.); mariano.mastinu@unica.it (M.M.); crnjar@unica.it (R.C.); 2 Obesity Surgical Unit ARNAS G. Brotzu, 09121 Cagliari, Italy; stepintuss@gmail.com; 3 Department of Life and Environmental Sciences, University of Cagliari, Monserrato, 09042 Cagliari, Italy; tcabras@unica.it

**Keywords:** taste sensitivity, salivary proteins, obesity, gender

## Abstract

Taste plays an important role in processes such as food choices, nutrition status and health. Salivary proteins contribute to taste sensitivity. Taste reduction has been associated with obesity. Gender influences the obesity predisposition and the genetic ability to perceive the bitterness of 6-*n*-propylthiouracil (PROP), oral marker for food preferences and consumption. We investigated variations in the profile of salivary proteome, analyzed by HPLC-ESI-MS, between sixty-one normal weight subjects (NW) and fifty-seven subjects with obesity (OB), based on gender and PROP sensitivity. Results showed variations of taste-related salivary proteins between NW and OB, which were differently associated with gender and PROP sensitivity. High levels of Ps-1, II-2 and IB-1 proteins belonging to basic proline rich proteins (bPRPs) and PRP-1 protein belonging to acid proline rich proteins (aPRPs) were found in OB males, who showed a lower body mass index (BMI) than OB females. High levels of Ps-1 protein and Cystatin SN (Cyst SN) were found in OB non-tasters, who had lower BMI than OB super-tasters. These new insights on the role of salivary proteins as a factor driving the specific weight gain of OB females and super-tasters, suggest the use of specific proteins as a strategic tool modifying taste responses related to eating behavior.

## 1. Introduction

The sensory properties of the food products, such as smell, sound, appearance and texture definitely influence what we select to eat. They cooperate in enabling individuals in long-range recognition of a potential food source [1]. However, taste acts as the final checkpoint and plays the most important role in processes such as food acceptance or rejection, eating preferences and choices, and therefore nutrition status and health [2,3,4,5,6,7,8,9,10]. 

In the mouth, before coming in contact with the receptor site of taste cells, chemical compounds of food are dissolved in saliva, whose variations of chemical–physical composition can contribute to individual variations of taste [11,12]. It is known that some salivary peptides belonging to PRP and histatin (Hist) families can bind polyphenols present in tannin-rich foods thus evoking the typical astringent sensation [13,14,15,16,17]. Salivary proteins play an important role in affecting sweet [18], salt [19], umami [20] taste, liking for fat and salt [21] and bitter acceptance [22]. Variations of the salivary proteome have been shown in human responses to bitter stimuli such as calcium nitrate, urea or quinine [23,24]. Our laboratory has been studying the involvement of salivary proteins in the ability to perceive the prototypical taste stimulus PROP [25,26,27,28,29,30]. We show that PROP super-taster and medium taster subjects, who perceive extreme and moderate bitterness from PROP, have higher basal levels of two basic PRPs (Ps-1 and II-2), compared to non-tasters who are taste blind to PROP. In addition, PROP stimulation increases the levels of the same proteins with respect to basal ones, only in PROP super-taster subjects. In addition, their supplementation, or that with amino acids, highly represented in the sequence of these proteins (i.e., L-Arg and L-Lys), enhances the sensitivity for PROP and for other taste qualities [26,27,31,32]. The proposed mechanism that describes the permissive role of these proteins or amino acids in taste perception, depending on their concentration in saliva, indicates that they could act as “carriers” of tastants, by increasing their solubility in saliva and thus the availability to taste receptor sites [27].

Individual variations of taste have been observed to be associated with various factors (i.e., genetics, environment, gender and age) and healthy outcomes [2,4,5,6,8,33,34,35]. Taste reduction, or reward from palatable food intake, can lead to compensatory strategies in response to energy dense foods, thus predisposing to higher risk of morbidity [36], metabolism disorders [37], worsening nutritional status [38], overweight and obesity [7,39,40,41,42,43,44,45,46,47,48,49,50]. Peculiarly, the ability to taste PROP, which is a human genetic trait, has been described as an oral marker for individual differences in general taste perception, food preferences and dietary behavior with consequent associations with body mass composition [2,3,4,51,52,53,54,55,56]. Several studies have shown a direct correlation between PROP sensitivity and fat perception [54] and PROP non-tasters to have a higher preference and shown lower discrimination for fat in food, compared to PROP super-tasters [52,53,54,56]. These findings support data showing that PROP perception is inversely correlated to calorie consumption [57,58], and that normo-weight non-tasters have a higher body mass index, with respect to normo-weight super-tasters [37,56,57,58,59,60,61,62]. However, this relationship has been observed to be reversed in obese subjects [37]. Several studies reported a gender effect on the ability to taste PROP [57,63,64] and on prevalence for obesity [65]. Although these differences might be due to hormonal factors, the reasons that drive them are not well understood [65]. Individual variations of taste as a function of age have been well-described [66]. Specifically, PROP sensitivity declined gradually and constantly with age [34,67]. These variations probably reflect well shown decreases in papillae density and functionality that occur with the aging process [68,69,70] and presumably contribute to changes in food preferences that occur with advancing age.

Based on these considerations it is evident that the salivary proteome composition in the oral cavity plays a key role in determining individual taste variations and these in turn are determinants to drive possible unbalanced food habits, which could lead to obesity. In this work, we evaluated whether normal weight (NW) subjects and subjects with obesity (OB) showed different profiles of basal levels (unstimulated saliva) of salivary proteins, also based on their gender and PROP sensitivity. 

## 2. Results

### 2.1. Effect of Gender and PROP Taster Status on BMI

**Figure 1:**
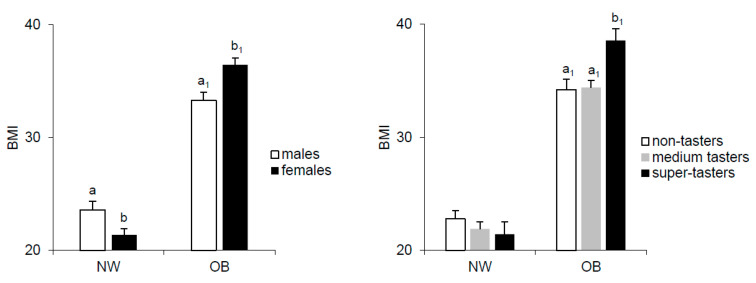
Mean values (± SEM) of body mass index (BMI) in normal weight (NW) (*n* = 61) and obesity (OB) (*n* = 57) according to gender (NW: male *n* = 21; female *n* = 40. OB: male *n* = 23; female *n* = 34) and to PROP taster status (NW: NT *n* = 22; MT *n* = 29; ST *n* = 10. OB: NT *n* = 14; MT *n* = 32; ST *n* = 11). a,b = significant difference within NW; a_1_,b_1_ = significant difference within OB (*p* ≤ 0.017; Fisher’s least significant difference (LSD) test.

Figure 1 shows mean values (± SEM) of body mass index (BMI) in NW and OB according to gender and PROP taster status. ANOVA revealed a significant two-way interaction of gender × NW/OB status (F_[1,14]_ = 17.142; *p* < 0.00007) and of the PROP taster status × NW/OB status (F_[2,112]_ = 4.713; *p* = 0.011) on BMI values. Post hoc comparison showed that the BMI of NW males was significantly higher than that of NW females, while the BMI of males was lower than that of females in OB (*p* ≤ 0.017; Fisher’s least significant difference (LSD) test. Post hoc also showed that the BMI of super-taster OB was significantly higher than that of medium taster and non-taster OB (*p* ≤ 0.002; Fisher’s test LSD). No significant difference in BMI related to PROP taster status was found in NW, although an opposite trend was evident (*p* > 0.05).

### 2.2. Salivary Protein Analysis

**Figure 2:**
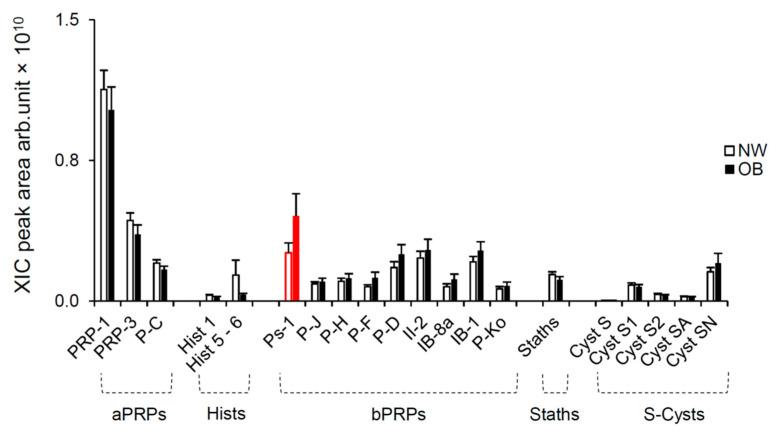
Mean values (±SEM) of the extracted ion current (XIC) peak areas of the PRP-1, PRP-3, P-C, Hist 1, Hist 5-6, Ps-1, P-J, P-H, P-F, P-D, II-2, IB-8a, IB-1, P-Ko, Staths, Cyst S, Cyst S1, Cyst S2, Cyst SA and Cyst SN in unstimulated saliva of NW (*n* = 61) and OB (*n* = 57). Red color indicates a significant difference between NW and OB (*p* = 0.00079; Fisher’s test LSD).

By the HPLC-ESI-IT-MS analysis we found differences in the extracted ion current (XIC) peak areas of the proteoforms (PRP-1, PRP-3 and P-C) belonging to the family of aPRPs, the peptides (Hist 1 and Hist 5-6) belonging to the family of Hists, the proteoforms (Ps-1, P-J, P-H, P-F, P-D, II-2, IB-8a, IB-1 and P-Ko) belonging to the family of bPRPs, the Staths and the proteins (Cyst S, Cyst S1, Cyst S2, Cyst SA and Cyst SN) belonging to the family of S-Cysts in the unstimulated saliva of NW and OB. The mean values (±SEM) are shown in Figure 2. We found a significant difference in the levels of Ps-1 protein with OB showing higher levels than NW (*p* = 0.00079; Fisher’s test LSD subsequent to two-way ANOVA). It is interesting to note that several individuals of the two groups were lacking this protein. The Ps-1 protein was undetected in 19 NW and 23 OB (χ2 = 1.09; *p* = 0.30).

#### 2.2.1. Effect of Gender

**Figure 3:**
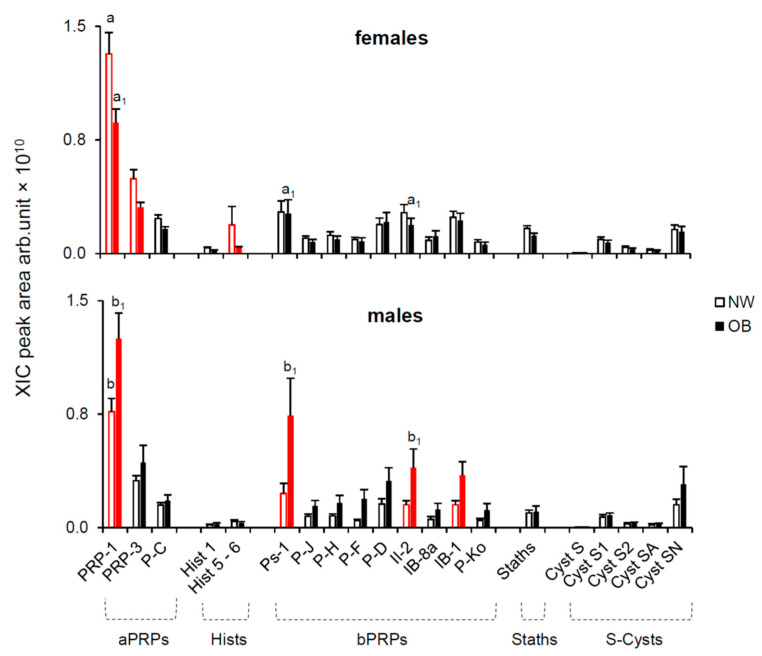
Mean values (±SEM) of the XIC peak areas of the PRP-1, PRP-3, P-C, Hist 1, Hist 5-6, Ps-1, P-J, P-H, P-F, P-D, II-2, IB-8a, IB-1, P-Ko, Staths, Cyst S, Cyst S1, Cyst S2, Cyst SA and Cyst SN in unstimulated saliva of NW and OB according to gender. Males: NW *n* = 21, OB *n* = 23; females: NW *n* = 34, OB *n* = 40. Red color indicates a significant difference between NW and OB (*p* ≤ 0.013; Fisher’s test LSD). a_1_, b_1_ = significant difference between females and males with obesity; a, b = significant difference between NW females and males (*p* ≤ 0.013; Fisher’s test LSD).

Figure 3 shows the mean values (±SEM) of the XIC peak areas of the same proteins belonging to the families of aPRPs, Hists, bPRPs, Staths and S-Cysts measured in unstimulated saliva of NW and OB males (lower graph) and females (upper graph). Three-way ANOVA revealed a significant interaction of gender × protein type × NW/OB status on the salivary protein levels (F_[20,2394]_ = 3.2908; *p* < 0.00001). Post hoc comparison revealed that unstimulated saliva of OB males had higher levels of PRP-1, Ps-1, II-2 and IB-1 than NW males (*p* ≤ 0.038; Fisher’s test LSD) (lower graph). While PRP-1 protein was detected in all male subjects, some were lacking Hist 5-6 (5 NW and 9 OB), Ps-1 (6 NW and 11 OB), II-2 (2 OB) and IB-1 (2 OB). However, no difference was found between NW and OB (*χ2* < 1.1716; *p* > 190). Differently, unstimulated saliva of OB females had lower levels of PRP-1, PRP-3 and Hist 5-6 with respect to NW females (*p* ≤ 0.042; Fisher’s test LSD) (upper graph). Post hoc comparison also showed that the levels of PRP-1, Ps-1 and II-2 of OB males were higher than those measured in OB females (*p* ≤ 0.013; Fisher’s test LSD) and the levels of PRP-1 in unstimulated saliva of NW males were lower than those measured in NW females (*p* = 0.000001; Fisher’s test LSD). The unstimulated saliva of all females had PRP-1 and PRP-3 proteins, while some were lacking Hist 5-6 (4 NW and 5 OB), Ps-1 (13 NW and 12 OB) and II-2 (1 NW and 3 OB). No difference was found between NW and OB females (*χ^2^* < 1.437; *p* > 0.231).

#### 2.2.2. Effect of PROP Taster Status

**Figure 4:**
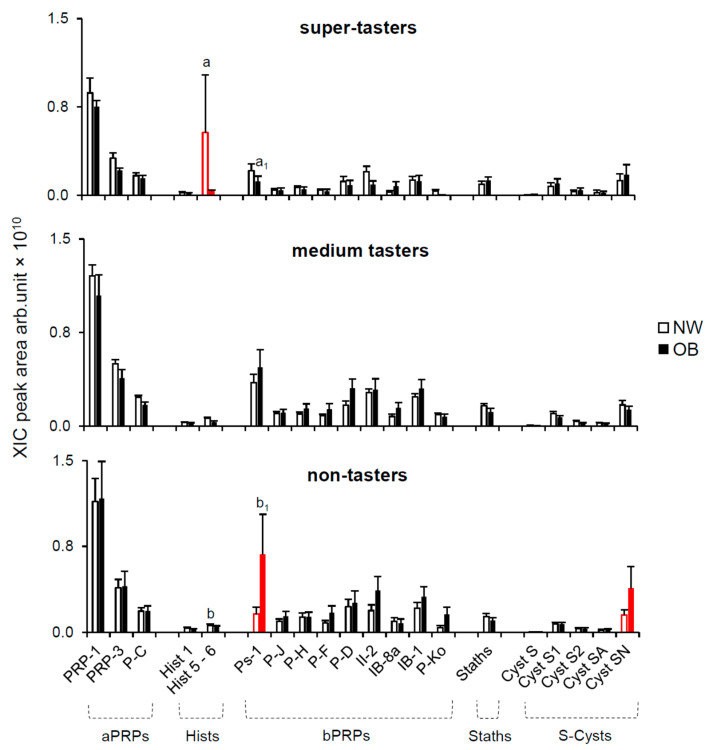
Mean values (±SEM) of the XIC peak areas of the PRP-1, PRP-3, P-C, Hist 1, Hist 5-6, Ps-1, P-J, P-H, P-F, P-D, II-2, IB-8a, IB-1, P-Ko, Staths, Cyst S, Cyst S1, Cyst S2, Cyst SA and Cyst SN in unstimulated saliva of NW and OB according to PROP taster status. Non-tasters: NW *n* = 22, OB *n* = 14; medium tasters: NW *n* = 29, OB *n* = 32 and super-tasters NW *n* = 10, OB *n* = 11. Red color indicates a significant difference between NW and OB (*p* ≤ 0.032; Fisher’s test LSD). a_1_, b_1_ = significant difference between OB super-tasters and non-tasters; a, b = significant difference between NW super-tasters and non-tasters (*p* ≤ 0.013; Fisher’s test LSD).

The mean values (±SEM) of the XIC peak areas of the same proteins belonging to the aPRPs, Hists, bPRPs, Staths and S-Cysts families measured in unstimulated saliva of NW and OB according to PROP taster status are shown in Figure 4. The XIC peak area of the Ps-1 and Cyst SN were significantly higher in the PROP non-taster OB, with respect to non-taster NW (*p* ≤ 0.032; Fisher’s test LSD subsequent to three-way ANOVA) and that of the Hist 5-6 was significantly lower in the PROP super-taster OB, with respect to super-taster NW (*p* ≤ 0.00039; Fisher’s test LSD subsequent to three-way ANOVA). No difference between NW and OB was observed in medium tasters (*p* > 0.05). OBs who were classified as non-tasters showed higher levels of Ps-1 than OBs who were super-tasters (*p* ≤ 0.000013; Fisher’s test LSD subsequent to three-way ANOVA). NWs who were classified as super-tasters had higher levels of Hist 5-6 than NWs who were non-tasters (*p* = 0.00009; Fisher’s test LSD subsequent to three-way ANOVA). Several NW and OB subjects of the PROP taster groups were lacking Hist 5-6 (super-tasters: 2 NWs and 3 OBs and non-tasters: 4 NWs and 3 OBs), Ps-1 (super-tasters: 2 NWs and 4 OBs and non-tasters: 9 NWs and 6 OBs) and Cyst SN (super-tasters: 1 NW and 2 OBs and non-tasters: 5 NWs). No difference was found between NW and OB super-tasters or non-tasters (*χ2* < 0.687; *p* > 0.47).

## 3. Discussion

**Figure 5:**
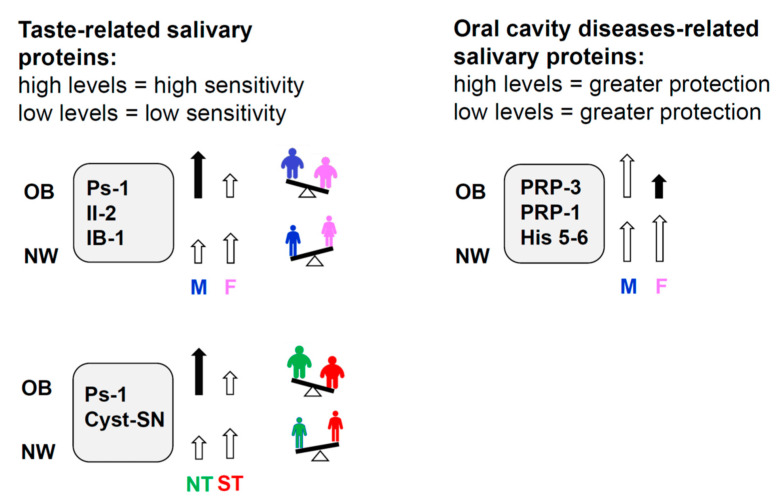
Picture showing a summary of findings on differences between NW and OB in salivary protein levels as a function of gender and PROP taster status. The height of arrows indicates the quantified level of proteins.

The primary aim of the present work was to investigate whether the salivary proteome composition, which can contribute to differences in taste perception [23,24,25,26,27,28,29,30], may be considered an important factor in driving unbalanced food behavior, which could lead to obesity. We showed, for the first-time, significant variations of taste-related salivary proteins, between NW and OB, which were differently associated with gender and PROP sensitivity. Interestingly, also BMI values were associated with gender and PROP sensitivity with an opposite trend in OB and NW. NW males showed higher values than NW females, while OB males showed lower values than OB females and, as already shown in previous studies [37,59], OB who were classified as super-tasters had a higher BMI than the other two PROP taster groups, while an opposite trend was found in NW [37]. A summary of principal results is shown in Figure 5.

HPLC-ESI-IT-MS analysis of unstimulated saliva of NW and OB allowed us to reveal that OB had higher levels of Ps-1 protein than NW. Since this protein and the amino acids highly represented in its sequence (L-Arg and L-Lys) have been shown to facilitate perception of hydrophobic molecules (i.e., PROP or oleic acid) by increasing their solubility in aqueous media [27,31], our finding seems to indicate that OB with higher levels of Ps-1 protein may find a greater reward in fat food intake, than NW.

The differences of salivary proteome that we found between OB and NW depend on gender: Ps-1, II-2 and IB-1, all proteins of bPRPs family, which had already been associated with higher taste perception [25], were lower in NW males than OB males and the latter had higher levels of Ps-1 and II-2 proteins than OB females. These results seem to indicate that a greater reward in response to palatable foods associated with obesity is specific for males. Differently, OB females who present lower levels of these proteins, and thus lower taste sensitivity, might be expected to eat more, as confirmed by their higher BMI. This result is consistent with data showing that a reduced oral perception strongly correlates with increased preferences for high-fat or energy foods [58], which lead to a higher consumption of these kinds of foods [56,57], a higher BMI [9,62,71] and a greater adiposity [61].

Our data also showed a strong effect of gender on the levels of PRP-1 and PRP-3 proteins of the aPRP family. It is known that PRP-1 and PRP3 have a strong affinity for the tooth mineral hydroxyapatite and can inhibit calcium phosphate precipitation, playing a role in the enamel pellicle formation, tooth protection and calcium homeostasis [72,73,74]. Previous studies also reported that peptides belonging to Hist family display an antifungal activity and inhibit bacterial enzymes involved in periodontal disease [75,76]. In addition, the occurrence of periodontitis was found to be more prevalent in OB females [77], who showed lower levels of Hist 5-6 than NW females. Our results showed that the levels of PRP-1, PRP-3 and Hist 5-6 were higher in NW than OB females, thus suggesting an increased probability of occurrence of caries and periodontitis in OB females according to studies that emphasize a high presence of dental disease in OB, especially in females [78,79,80]. The low levels of the PRP-1, together with the lower levels of Ps-1 and II- 2, that we found in OB females let us speculate that diseases in the oral cavity create an adverse environment for the dissolvement of food chemicals in saliva and for the interaction between tastants and taste receptors, eventually affecting overall taste perception.

The chemical interaction between taste stimuli and salivary proteins has been shown to explain the big phenotypic differences in the PROP taste perception [12,25,26,27]. Specifically, high levels of Ps-1 and II-2 proteins are associated with high PROP sensitivity, while low levels with low sensitivity. In addition, low sensitivity in NW is associated with higher preference and lower discrimination for foods high in calories [52,53,54] and this leads to an unbalance of food habits affecting nutritional status and BMI [57]. In addition, the oral supplementation of Ps-1 protein, and that of amino acids (L-Arg and L-Lys), has been shown to modify the perception of PROP and the five taste qualities [25,26,27,32]. Differently, in OB we found that the phenotype with low sensitivity showed higher levels of Ps-1 with respect to that with high sensitivity according to its higher BMI values. It is interesting to note that OB non-tasters also had higher levels of Ps-1 protein than NW non-tasters and this proves their higher sensitivity.

Noteworthy, proteins of the bPRP family are secreted in saliva exclusively by parotid glands [74,81] and gland activity is affected by gender and obesity status. Inoue et al. showed that young NW females have smaller parotid and submandibular gland sizes as compared to males [82]. Moreover, Bozzato et al. [83] described a deposition of adipocytes in parotid glands of OB, but not in submandibular glands, with a correlation between BMI and gland size. This allowed us to speculate that the bPRPs synthesis and secretion depend on gender and nutritional status.

Our results also show that OB who were classified as non-tasters had higher levels of Cyst SN than NW non-tasters. Hyposensitive subjects for the bitterness of caffeine express higher salivary levels of Cyst SN than hypersensitive subjects, and it was overexpressed in infants that accepted a bitter solution [22,84]. In vitro experiments on human submandibular gland cells showed an overexpression of Cyst SN after caffein stimulation [85] and in vivo experiments after cranberry juice, a model stimulus for astringency [16]. In addition, Cyst SN is overexpressed in sensitive subjects for oleic acid (C18:1) [86]. These considerations support the hypothesis that OB non tasters having high levels of Ps-1 and Cyst SN may have an increased taste sensitivity compared to NW non-tasters.

In conclusion, this work shows novel insights on the role of salivary proteome as a factor driving the greater propensity for body weight excess of females or that associated with higher PROP sensitivity, which have been already shown [37,65]. However, further studies should investigate the effect of the oral supplementation with Ps-1 protein or amino acid L-Arg on taste perception in OB to test the hypothesis that this mechanism may alter taste response related to foods for the development of a sex or taste-specific approach for weight loss treatment. In addition, the oral modification of specific salivary components might find application also by solving the increased predisposition for all diseases, which are related with unbalanced food habits in subjects with reduced taste [7,36,37,38,39,40,41,42,43,44,45,46,47,48,49,50,87].

## 4. Materials and Methods

### 4.1. Volunteers

One hundred and eighteen volunteers were recruited for the study through public advertisements in the area of the city of Cagliari (Italy). Volunteers were divided into two groups based on the body mass index (BMI), resulting in a random classification by gender and age: OB who had a BMI ranging from 30 to 50 kg/m^2^ (*n* = 57; females = 34, males = 23; age ranging from 19 to 72) and NW who had a BMI ranging from 18 to 25 kg/m^2^ (*n* = 61; female = 40, males = 21; age ranging from 20 to 67). Exclusion criteria for both groups included major diseases, otolaryngology disorders, pregnancy or lactation, food allergies, active caries and use of drugs that interfere with taste. OBs with metabolic disorders were also excluded. Prior the enrollment in the study, the study protocol was verbally explained to OB and NW who signed an informed consent form.

### 4.2. Experimental Procedure

The study was conducted in one session. Trials took place in the morning in a controlled environment condition (23 °C; 40–50% of humidity). Volunteers were asked to abstain from eating, drinking except water and using oral care product at least 8 h prior to the saliva sample collection and the screening test for PROP taste sensitivity. They were required to be in the test room 15 min before the beginning of the session. Firstly, weight (kg) and height (m) were recorded in order to calculate BMI (kg/m^2^). Subsequently, a sample of whole unstimulated saliva was collected from each volunteer in an Eppendorf tube (1 mL) for proteins quantification as described below. Finally, volunteers were classified for their PROP taster status by using the impregnated paper screening test [88], which has been tested for validity and reliability [16,30,88] and strongly correlates with the taste cell depolarization [89]. This test requires evaluations of perceived taste intensity for two paper disks (one impregnated with PROP solution; 50 mmol/L; Sigma-Aldrich, Milan, Italy and the other with NaCl, 1.0 mol/L; Sigma-Aldrich, Milan, Italy) by using the label magnitude scale (LMS). Volunteers who rated PROP disk lower than 15 mm were classified as PROP non-tasters, those who rated PROP disk higher than 67 mm were classified as PROP super-tasters. Subjects who rated PROP disk between 15 and 67 mm were classified as PROP medium-tasters. Ratings for NaCl disk were used to classify volunteers who gave borderline ratings for PROP disk.

### 4.3. Salivary Protein Analysis

The three proteoforms (PRP-1, PRP-3 and P-C) belonging to the family of aPRPs, the three peptides (Hist 1, Hist 5 and Hist 6) belonging to the family of Hists, the nine proteoforms (Ps-1, P-J, P-H, P-F, P-D, II-2, IB-8a, IB-1 and P-Ko) belonging to the family of bPRPs, the proteoforms belonging to the family of Staths (di-phosphorylated, mono-phosphorylated and non-phosphorylated) and the five proteoforms (Cyst S, Cyst S1, Cyst S2, Cyst SA and Cyst SN) belonging to the family of S-Cysts were identified and quantified in each saliva sample, by high performance liquid chromatography-electrospray ionization-ion trap-MS (HPLC-ESI-IT-MS) (ThermoFisher, San Jose, CA, USA) according to our previous studies [16,17,25,26,27]. The measurements were performed by a Surveyor HPLC system connected by a T splitter to a diode-array detector and to an LCQ Advantage mass spectrometer provided with an ESI source (ThermoFisher Scientific, San Jose, CA, USA). Saliva samples were treated with trifluoroacetic acid (0.2%; Sigma-Aldrich, Milan, Italy) according to [25] immediately after collection. Salivary peptides and proteins were analyzed using the HPLC-Low Resolution-ESI-IT-MS technique following Cabras et al. 2012 [25]. Briefly, 30 µL of acidic soluble fraction corresponding to 15 µL of whole unstimulated saliva were used. Average mass values (Mav), obtained by deconvolution of averaged ESI-MS spectra automatically performed by using MagTran 1.0 software (Amgen Inc., Thousand Oaks, CA, USA) [90], and elution times of proteins/peptides were compared with those determined in our previous studies [81,91] and with the theoretical ones available at the UniProtKB/Swiss-Prot human database (http://us.expasy.org/tools). The quantification of each protein and peptide was based on the area of the HPLC-ESI-IT-MS extracted ion current (XIC) peaks. The XIC analysis revealed the peak related to the proteins of interest by searching, along the total ion current chromatographic profile, the specific multiply charged ions generated by the protein. The area of the ion current peak is proportional to the concentration, and under constant conditions it may be used to perform relative quantification of the same analyte in different samples [92]. Since the XIC peak areas of the His-5 and His-6 peptides, and those of the three proteoforms of the family of Staths, showed the similar trend in all subjects, they were considered and analyzed as a sum, and shown in the figures as Hist 5-6 and Staths, respectively.

### 4.4. Statistical Analyses

Differences in BMI between NW and OB according to gender and PROP taster status were analyzed by using a two-way ANOVA. A two-way ANOVA was also used to compare differences in the basal levels of PRP-1, PRP-3, P-C, Hist 1, Hist 5-6, Ps-1, P-J, P-H, P-F, P-D, II-2, IB-8a, IB-1, P-Ko, Staths, Cyst S, Cyst S1, Cyst S2, Cyst SA and Cyst SN in NW and OB. Three-way ANOVA was used to evaluate differences in protein levels related to gender and PROP taster status in NW and OB. Post hoc comparisons were performed with the Fisher’s least significant difference (LSD) test. The Fisher’s exact test was used to compare the number of NW and OB subjects lacking a protein. *p* values ≤ 0.05 were considered significant. Statistical analyses were conducted with STATISTICA for WINDOWS (version 10; StatSoft Inc., Tulsa, OK, USA).

## Data Availability

The data presented in this study are available on request from the corresponding author. The data are not publicly available in accordance with consent provided by participants on the use of confidential data.

## Sample Availability

Samples of the compounds are available from the authors.
